# Medical and physiological complications of exercise for individuals with an eating disorder: A narrative review

**DOI:** 10.1186/s40337-022-00685-9

**Published:** 2023-01-10

**Authors:** Danika A. Quesnel, Marita Cooper, Maria Fernandez-del-Valle, Alanah Reilly, Rachel M. Calogero

**Affiliations:** 1 Department of Psychological Clinical Science, University of Toronto, 320 Huron Street, Toronto, ON M5S 3J7 Canada; 2 Department of Psychiatry and Behavioral Sciences, Children’s Hospital of Philadelphia, Philadelphia, PA USA; 3 Department of Functional Biology, University of Oviedo, Oviedo, Spain; 4 Health Research Institute of the Principality of Asturias (ISPA), Oviedo, Spain; 5 Alanah Reilly Exercise Physiologist, Brisbane, Australia; 6 Psychology Department, Western University, London, ON Canada

**Keywords:** Eating disorders, Dysfunctional exercise, Physical activity, Exercise, Incidental activity, Exercise physiology, Treatment

## Abstract

Individuals with eating disorders (ED) experience prolonged malnutrition, binge episodes, and compensatory behaviours that affect every organ system. Psychological and physiological symptoms are worsened with comorbid dysfunctional exercise, seen in up to 80% of those with an ED. Although return to exercise is an important component of treatment and recovery, little is known about the contraindications and risks of exercise engagement specific to those with an ED. This paper provides a comprehensive narrative review of the medical and physiological complications of engaging in exercise during ED treatment and outlines when exercise may be contraindicated or used in modified or cautionary ways. We conducted a literature search on MEDLINE, PubMed, and PsychArticles to identify relevant articles, which yielded six categories of medical and physiological complications of ED that may be exacerbated by exercise: energy availability, cardiovascular health, electrolyte abnormalities, biomedical function markers, sex hormones, and body composition. We summarize the evidence for these complications for readers and offer an initial set of recommendations for incorporating exercise during ED treatment based on our findings. This review may serve as a resource for members of ED treatment teams to help evaluate more readily and confidently whether exercise is safe for individual patients and when modifications and caution may be warranted.

## Plain English summary

Dysfunctional exercise (DEX) is a symptom of eating disorders (ED) that precedes, maintains and exacerbates ED pathology. Health professionals struggle to clinically address and manage DEX as little information is available about its assessment and safe management. The current review provides a comprehensive summary of the medical and physiological complications of ED that may be exacerbated by exercise and outlines when exercise may be contraindicated or used in a modified or cautionary way. The literature review yielded six categories of complications: energy availability, cardiovascular health, electrolyte abnormalities, biomedical function markers, sex hormones, and body composition. We summarize the evidence for these complications for readers and offer an initial set of recommendations for incorporating exercise during ED treatment based on our findings. This review may serve as a resource for members of ED treatment teams to help evaluate more readily and confidently whether exercise is safe for individual patients and when modifications and caution may be warranted.

## Introduction

Eating disorders (ED) are serious psychiatric illnesses with a lifetime prevalence of 10% [1]. Prolonged malnutrition, binge episodes, and purging in EDs affect every organ system [2]. Beyond physiological complications, individuals with ED exhibit high rates of comorbid disorders, suicidality, and decreased quality of life [3–7]. Although many medical complications resolve with adequate nutrition and treatment, mortality rates in ED populations remain high and relapse is unfortunately common [1, 8]. Those with an ED who also engage in dysfunctional exercise are more likely to experience poor physical and psychological health outcomes [9, 10]. We define dysfunctional exercise as a pathological relationship with exercise resulting in negative physical and/or psychological health impairment; an umbrella term encompassing compulsive exercise, exercise addiction, obligatory exercise, and exercise dependence [11–13]. Dysfunctional exercise is present in 22–80% of individuals with an ED [9]. Acutely, these individuals may experience more severe psychological distress, worsened ED psychopathology, and poorer health-related quality of life; and chronically, greater rates of depression, less satisfaction with life, greater risk of relapse, require a longer treatment duration, and exhibit an enduring course of illness [10, 14–18].

Given the potential for dysfunctional exercise to exacerbate ED pathology and undermine recovery efforts, many practitioners recommend abstinence from exercise during ED treatment [19]. However, this practice has been increasingly questioned [20–24], bringing with it a shift toward incorporating physical activity during ED treatment [17, 25, 26]. Similar to the importance of restoring normative eating behaviors and cognitions during treatment, restoring a normative relationship with physical activity may also be vital to recovery, whether through formalized exercise (e.g., strength training, cycling, yoga), incidental activity (e.g. folding laundry, grocery shopping), or a combination of the two [12]. Despite initial concerns about the impact of exercise on weight restoration outcomes, data have not revealed any differences between those who engage in supervised exercise during ED treatment and those who do not [10, 23, 27, 28]. Conversely, supervised and therapeutic exercise including yoga [29, 30], strength training [31–35] endurance activities, [36], outdoor games/sports [37, 38] during ED treatment appears to predict improved physical and mental health outcomes for individuals living with an ED [10, 11, 18, 25, 26, 28, 31, 35, 39]. Benefits of exercise interventions reportedly include improvements in clients’ body composition [31, 32], fitness markers such as VO2max [36], DEX markers [26, 39] quality of life [33, 37], therapeutic alliance [40] and drive for thinness [29] amongst other benefits. Considering exercise compulsion at discharge from treatment predicts a shorter time to relapse and poorer overall outcomes [16], there is a critical need to promote safe, therapeutic, evidence-based, and nutritionally supported exercise during ED treatment, as it accounts for an important part of treatment.

Despite the promising outcomes of safe exercise interventions during ED treatment, EDs are associated with serious medical comorbidities that may contraindicate specific exercise modalities, intensities, or amounts. Thus, prior to initiating an exercise program with an individual with an ED, we must understand the contraindications and risks of exercise engagement specific to this population. This paper provides a comprehensive narrative review of the medical and physiological complications of engaging in exercise and outlines when exercise may be contraindicated.

## Methods

To identify relevant empirical articles for this review, we conducted a literature search on MEDLINE, PubMed, and PsychArticles using the following keywords as our search terms: “eating disorder* OR anorexi* OR bulimi*” AND “exercise OR physical therapy OR physical activity” AND “cardiorespiratory OR mental OR metabolic OR neurological OR musculoskeletal OR mortality OR adverse events OR injury.” All articles were published articles or dissertations of which the samples included individuals with eating disorders or disordered eating behaviors. We did not define inclusion and exclusion criteria, although we did exclude studies with youth (< 18 years) and athlete only populations, as we considered the unique needs of these groups as beyond the scope of this review. Based on review, we categorized content into different themes.

## Results

### Physiological complications of exercise with an eating disorder

Table 1 summarizes the medical and physiological complications of exercise with ED. We organized the complications under six categories: Energy Availability, Cardiovascular Health, Electrolyte Abnormalities, Biomedical Function Markers, Sex Hormones, and Body Composition. For each complication included in Table 1, we provide a definition, the values of concern related to it, and the bodily systems (e.g., cardiovascular, endocrine) affected by it with ongoing exercise engagement. Given that an additional purpose of this review was to identify risks and recommendations related to exercise engagement for individuals living with EDs, we excluded complications and conditions related to ED (e.g., hyperlipidaemia, polycystic ovary syndrome, and nonalcoholic fatty liver disease) that appear to be linked to positive benefits of exercise without necessary modifications [41–43]. Further, we have not included details on common comorbid diagnoses of ED (e.g., Type 1 and 2 diabetes, chronic pain, and asthma) [43–47] as reviews and clear guidance exist for exercising with these conditions [48–51]

**Table 1:** Summary of complications of an eating disorder exacerbated by exercise and their impact on the overarching systems of the body

Complication	Definiton	Value	Endo	Gastro	Cardio	PSYC	HEMA	MET	MENSI/REPRO	Bone	IMMU	MUSC	RESP	OTHER
*Energy availability*														
Low energy ayailability	LEA occurs when an individual’s current energy intake does not adequately cover both exercise and physiological requirements	*OEA is ≥ 45 kcal/kg of FFM/day or 188 kj/kg of FFM/day *LEA is < 30 kcal/kg (125kj/kg) of FFM/day*	X	X	X	X	X	X	X	X	X			
Starvation	Low energy availability including dieting and ‘cleanses’	< 30 kcal/kg of FFM/day for females, < 15 -30 kcal/kg of FFM/day for males			X		X					X		
Purging	Self-induced vomiting, misuse of laxatives or diuretics	Fluid losses of up to 5000 mL			X	X	X			X		X	X	Hypohydration electrolyte loss
*Caridovascular health*														
Arrythmia	Change in the normal sequence of electrical impulses causing an abnormal heartbeat rhythm				X									Organ damage
Bradycardia	Low resting heart rate	< 60 bpm			X	X							X	Fainting
Tachycardia	High resting heart rate	> 100 bpm			X							X		Fainting
Postural tachycardia	An excessive heart rate increase upon standing	↑ ≥ 30 bpm within the first 10 s of standing from lying			X	X						X	X	Neurology deficits, blurred vision, coldness
Orthostatic hypotension	Sustained drop in systolic or diastolic blood pressure	Systolic drop of ≥ 20 mmHg or diastolic pressure drop of ≥ 10 mmHg after 3 min of standing		X	X									Fainting, blurred vision
Hypotension	Low resting blood pressure	< 90/60 mmHg		X	X	X							X	Dizziness, lightheadedness fainting, cold, blurred vision
Hypertension	High blood pressure	> 140/90 mmHg			X									Head aches
Prolonged QTc	Electrical heart current abnormality, the time beginning of the Q wave to the end of the T wave on an ECG	> 460 ms in women and > 450 ms in men			X									
*Electrolyte abnormalities*														
Hypokalemia	Low serum potassium	< 3.6 mmol/L (64.8 mg/dL), severe hypokalemia < 2.5 mmol/L (45 mg/dL)			X							X	X	Constipation, paralysis
Hypophosphatemia	Low serum phosphate	< 0.8 mmol/L (1.46 mg/dL)		X	X	X	X				X	X	X	
Hypomagnesemia	Low serum magnesium	< 0.081 mmol/L(1.46 mg/dL)	X		X	X						X	X	Electrolytes, tetany vertigo, impaired vision, seizures
Hypercarbia	Metabolic alkalosis or high blood serum bicarbonate level	> 30-40 mmol/L (540 mg/dL – 720 mg/dL)												
Hyponatremia	Low concentration of serum sodium	Mild to moderate < 20-130 mmol/L, or severe < 120 mmol/L		X	X	X								Seizures
*Biomedical function markers*														
Hypothermia	Low body temperature	< 35 °C or < 95°F			X	X						X		
Hyperthermia	High body temperature	> 36 °C or > 96.8°F												Heat stroke
Blood urea nitrogen (BUN) & urine specific gravity (USG)	BUN: Test measuring urea in the blood USG: Urinalysis parameter to measure kidney function	BUN: 1.010 to 1.030 Low USG: 1.001–1.003												Low kidney & adrenal functioning
Transaminase	Enzymes released into the blood due to liver damage	3X blood aminotransferase and alanine aminotransferase			X									Indicates vital organ failure
Hypoglycemia	Decreased liver and circulating glycogen	Blood glucose level > 70 mg/dL < 4 mmol/L										X		Headaches, brain damage
Hypohydration	Low hydration	Loss of 1–3% of body weight in water		X	X							X		
*Sex hormones*														
Amenorrhea/ functional hypothalamic amenorrhea	Absence of menstrual cycles, FHA is a type of amenorrhea related to LEA	No cycle > 90 days	X				X		X	X		X		
Female sex hormones	GnRH, LH, estrogen, FSH	_	X				X		X	X		X		
Male sex hormones	Testosterone, LH, FSH, GnRH, as well as abnormal LH pulsality	_	X				X			X	X	X		
*Body composition*														
BMI^†^	Ratio of weight to height	18.5 – 24.9 kg/m^2^	X		X	X		X		X	X	X		
Bone mineral density—males	Amount of bone mineral related to bone tissue	Low BMD = Z-score less than -1.0								X				
Bone mineral density—females	Amount of bone mineral related to bone tissue	Low BMD = Z-score less than -1.0								X				
Superior mesenteric artery syndrome	Compression of the duodenum due to a diminished fat pad	N/A		X										Abdominal pain
Peripheral edema	Fluid swelling of extremities	N/A			X									Indicates cardiac failure

*Threshold for OEA and LEA are guidelines only and differ across individuals and sex. Energy needs also increase relative to physical activity demands. ^†^Although a “healthy” BMI range is often classified as 18.5-24.9 kg/m^2^, this needs to be considered within an individual’s premorbid weight and historical growth curve. ENDO: endocrine functioning; GASTRO: gastrointestinal functioning; CARDIO: cardiovascular functioning; PSYC: impact on psychological functioning; DEVEL: impairment of growth and development; HEMA; Hematocrit abnormalities; MET: Metabolic functioning impairment; MENSI; Menstrual Functioning and Reproduction implications; MUSC; Musculture: BONE; implications for bone health; IMMU; Immune functioning; RESP; respiratory system implications; LEA: low energy availability; OEA: optimal energy availablility; RED-S: Relative Energy Deficiency in Sport; kcal/kg: kilocalories per kilogram; kj/kg: kilojoules per kilogram; FFM: fat free mass; mL: millilitres; mmHg: millimetres of mercury; bpm: beats per minute; ms: milliseconds; mmol/L: millimoles per liter; mg/dL: milligrams per deciliter; °C: degrees Celsius; °F: degrees Fahrenheit; %: percent; FHA: functional hypothalamic amenorrhea; GnRH: Gonadotropin-releasing hormone; LH: luteinizing hormone; FSH: follicle-stimulating hormone; kg/m^2^; kilograms per meter squared; BMD: Bone mineral density; *: this is an advisory cut off, not definite.

## Energy availability

### Energy deficiency

Optimal energy availability (OEA) is defined as adequate energy intake given an individual’s typical metabolic functioning and level of exercise engagement. OEA is commonly estimated as at least 45 kilocalories (kcal) (188 kilojoules; kj) per kilogram (kg) of fat free mass (FFM) per day (45 kcal/kg of FFM/day) [52, 53]. This intake provides adequate energy availability for the human body and its systems to thrive, after subtracting the energy needed and used for regular exercise and recovery. Increased energy demands due to physical activity will likely require more than this standard amount. Low energy availabity (LEA) occurs when: 1) energy intake is insufficient to support vital bodily functions (after removing the energy cost of exercise relative to metabolically active FFM) or 2) exercise engagement increases without an adequate increase in energy to meet heightened energy output needs [54]. Importantly, the numerous and detrimental effects of LEA can occur irrespective of an individual’s body size, shape or weight and does not solely occur in those with ED [55].

Initially, LEA impacts the body’s ability to engage in, and recover from, physical activity by hindering the adequacy of glycogen stores and protein synthesis [56]. If LEA persists, the body is forced to adapt and conserve energy wherever possible by no longer performing non-essential functions such as regular menses, hair growth, mood regulation, or executive thinking, as well as reducing cellular metabolism, weakening the potency of the immune system, and decreasing cardiovascular capacity [57] This conservation process reduces both absolute and relative resting metabolic rates (RMR) to protect the unfavourable breakdown of FFM (metabolically active protein tissue including muscles, bones and organs; [58]. This reduction in RMR can negatively impact both health and performance outcomes [59]. If energy availability falls below optimal levels, metabolically active tissue can breakdown to provide the vital body systems with sufficient energy for survival, leading to even greater decreases in RMR [59]. Even the cardiac muscle may be used to produce this energy, with muscle mass and visceral adipose tissue also beginning to atrophy. Other negative consequences, such as psychological distress [60] can also occur during this LEA state.

Despite the serious consequences of LEA, there is not yet a standardised, reliable and valid assessment protocol for measuring energy availability [59]. Current measurement approaches require specialised equipment and expertise and are burdensome, requiring individuals to keep an excessively accurate food and execise log [59]. Adding to this complexity, exercise output often changes during different levels of activity engagement [59]. Despite these measurement difficulties, LEA contributes to additional conditions described in subsequent sections of the article that may either contradict exercise, worsen performance, and/or endanger health.

### Starvation

Starvation occurs when there is a severe deficiency in caloric intake below the level needed to maintain an organism. Starvation can result in a loss of cardiac mass (particularly ventricular mass) along with glycogen depletion, and BMD causing a premature reduction in physical and physiological capacity [61]. Exercising in a starved state encourages circulatory lactate producing widespread muscular pain [61] and, with dehydration, may trigger muscular cramps due to lack of sodium and potential magnesium. Individuals who are undernourished while engaging in intense exercise programs may trigger loss of FFM (including cardiac mass), provoking a reduction in muscle strength and aerobic performance [lower maximal oxygen uptake (VO_2_max), decreased work capacity] and EKG changes with exercise testing [61]. Starvation is not necessarily a direct contraindication for exercise engagement; however, for those with an ED, exercise may be contraindicated on days when they have intentionally skipped a meal [62]. Additionally, continuous difficulty in meeting one’s nutritional plan may necessitate modifying exercise, due to the self-reinforcing cycle of restriction and exercise[63]

### Purging

Purging is a compensatory behaviour (e.g., self-induced vomiting, laxative or diuretic abuse) to “get rid” of food or calories [64]. All methods of purging detrimentally affect health and exercise performance and are associated with serious medical risks. Mehler and Andersen [65] describe three mechanisms through which purging can influence exercise engagement: 1) the generation of a negative caloric balance (through vomiting); 2) the facilitation of dehydration (vomiting and laxative use); and 3) the contribution to hypokalemia (vomiting and laxative use) [66]. Purging (vomiting and laxative use) contributes to critical electrolyte disturbances and water loss, which are both exacerbated by exercise due to the natural loss of electrolytes and water in sweat [65, 67, 68]. Purging may also lead to hypovolemia (decreased blood plasma volume), which can contraindicate exercise [65]. Medical risk and complications differ across modes of purging and compensatory behaviors (e.g., exercise) and starvation. This can be attributed to the concentration of acid and electrolytes in the relevant location and impact of volume loss; for example, vomitus contains a high concentration of acid and potassium [69]. We also observe differences in the mechanisms of action among common laxative classes, resulting in side effects ranging from bicarbonate resorption, expulsion of sodium, or potassium loss [70]. These differences are observed in different rates of electrolyte imbalances across patients with ED, e.g., combined low BMI, laxative use, and vomiting yields the highest likelihood of developing hypokalemia, vomiting related to hypomagnesemia, and fasting or exercise relatively unrelated to potassium levels [71, 72].

### Cardiovascular health

LEA contributes to many cardiovascular complications in individuals with an ED including arrhythmias, bradycardia, tachycardia, EKG abnormalities, and orthostatic vitals. Additionally, several cardiac risk markers including mean R and T wave amplitude may be abnormal in those with an ED [73–75]. The following section provides an overview of cardiovascular impacts of unmodified exercise for individuals with an ED.

### Hypotension

Hypotension is defined as a low blood pressure (< 90/60 mmHg), where blood does not exert necessary pressure on the artery walls [65, 76]. This pressure is required to supply vital organs with adequate oxygen and energy to function; thus, low blood pressure contributes to inadequate oxygen and nutrient availability, compromising vital organ functioning [77]. Low blood pressure in individuals with an ED may occur due to purging or insufficient food/fluid intake, resulting in circulating blood volume depletion [78] or autonomic dysfunction related to disturbances in key hormones and/or other mechanisms regulated by the central nervous system [79]. Even mild cases of dehydration (1–2% of body weight) can cause an individual to become symptomatic [76]. Lastly, hypotension can result from low electrolyte concentration, resulting in low concentration gradient and subsequent loss of circulating blood volume [68]. In the case of hypotension, its recommended for exercise engagement to be modified to mitigate dehydration [78].

### Hypertension

Hypertension, also known as high blood pressure (> 140/90 mmHg) is a condition in which the blood vessels experience persistently raised pressure against them [80]. Over time, hypertension can damage arteries, prevent blood and oxygen supply to the brain, heart, and body, result in heart enlargement, prevent kidneys from filtering blood effectively, or arterial function (including the small vessels inside the eyes) [81]. Bulimia nervosa and BED have higher rates of hypertension compared to other EDs [82]. Although exercise can be protective against progression of hypertension and cardiovascular mortality risk, exercise for individuals with hypertension may require modification, medical supervision, or at times may be contrinadicated.

Additionally, rates of idiopathic intracranical hypertension in BED are also elevated [83], which can lead to intracranial pressure and increased risk of stroke. When these individuals engage in exercise it may lead to headaches [84]. Despite this, contraindications to any type of exercise have not been proposed [80]. However, the incidence of hypertension onset has been experienced by those who engage in high volumes of exercise, which could be exacerbated by a lack of energy and nutrition [85]. Although exercise is often recommended for those with high blood pressure (140/90 mm/Hg), individuals with very high blood pressure (> 200 mm/Hg) may require pharmacological intervention and medical clearance prior to exercise.

### Arrhythmias

Arrhythmias refer to any change from the normal sequence of electrical impulses within the heart resulting in an abnormal heartbeat rhythm [86]. For an individual with an ED, arrhythmias may result from LEA [87], malnutrition, and/or hypokalemia [88]. Arrythmias occur when dietary intake is inadequate to support both vital function and exercise engagement, relative to metabolically active FFM [59]. Arrhythmias can present, and persist, irrespective of weight status [87]. Symptoms of arrhythmia may include fatigue, weakness, dizziness, light-headedness, fainting or near fainting, rapid heartbeat, shortness of breath, anxiety, chest pain or pressure, and in extreme cases, collapse, syncope and cardiac arrest [86]. These symptoms may require close monitoring for those with ED including both EKG and Holter monitoring. The results of exercise testing by a professional can determine the safe duration, type, and intensity of exercise.

### Bradycardia

Bradycardia is a low resting heart rate (< 60 bpm) and is a subtype of arrhythmia [89], often seen in AN [78]. The specific physiological cause of bradycardia is unclear [65] However, hypothesized causes of bradycardia include increased parasympathetic tone and loss of cardiac mass, [90, 91], vagal hyperactivity, [92], and LEA [87], in an effort to decrease strain on the heart by reducing cardiac output. Bradycardia can present, and persist, irrespective of weight status [87] and symptom severity is associated with greater frequency of exercise engagement and team sport engagement [90, 91].

Gibbons et al. [93] warn that sustained arrhythmia can interfere with cardiac output responses during exercising and, in particular, that bradyarrhythmias (with the potential to become more complex or to impede hemodynamic stability) may contraindicate exercise [93]. Fletcher et al. [94] add that uncontrolled cardiac arrhythmias with hemodynamic compromise may contraindicate exercise engagement [94]. The American College of Sports Medicine [95] recommends any noticeable change in heart rhythm by palpitation or auscultation may contraindicate exercise [95]. Likewise, McCallum et al. [78] suggest individuals experiencing an ED with bradycardia and purging should only engage in supervised exercise, and only when nutritional intake and electrolyte and fluid levels are adequate, particularly when under 80% of target body weight.

### Tachycardia

Tachycardia, a second subtype of arrhythmia, is a high resting heart rate of > 100 bpm [96]. Tachycardia in AN may indicate more severe autonomic nervous dysfunction than bradycardia and suggests a greater likelihood of poor long-term outcomes [97]. Tachycardia comprises several subtypes, ranging from a natural stress response, to abnormal, dangerous responses indicating a fault in the heart’s electrical system [96]. The latter may involve the heart beating faster than chambers can refill with blood, ultimately compromising vital blood flow to the rest of the body and potentially causing death [96]. In those with an ED, tachycardia must be carefully investigated prior to any exercise engagement.

### Postural tachycardia

Orthostatic changes, as in postural tachycardia and orthostatic hypotension, represent the change between vital sign readings during the initial minutes of standing or upright tilt from the original lying position [78, 98]. Postural tachycardia is when a heart rate increases of ≥ 30 bpm (≥ 40 bpm in those aged 12–19 years old) persists for more than 30s [99, 100]. In a typical response to an upright posture change, the peripheral veins constrict and an increase in heart contractility occurs [101]; however, when these changes do not occur, blood pools in the peripheral parts of the body, causing the heart to beat abnormally fast in an effort to return sufficient blood to the brain [99]. This compensatory effort is crucial to ensure constant oxygen supply to the brain [99]. Central nervous system symptoms, a complete loss of consciousness, or even sudden cardiac death can ultimately occur if too much blood remains pooled in the extremities, as the brain becomes starved of oxygen and nutrients [100]. Postural tachycardia may result from LEA, [60], autonomic nervous dysfunction [102] and give way to weakness or postural dizziness [78], decreased heart muscle mass (particularly the left ventricle), decreased stroke volume, decreased blood volume [103] and emotional stress [104]. Postural tachycardia mimics the cardiac symptoms of Postural Orthostatic Tachycardic Syndrome; however, this syndrome should not be diagnosed in the presence of malnourishment [60]. Typically, postural tachycardia resolves as weight and nutritional status improve, suggesting that exercise may be gradually modified with symptom improvement[78].

### Orthostatic hypotension

Orthostatic hypotension is defined as a sustained drop in systolic blood pressure of ≥ 20 mmHg or diastolic pressure of ≥ 10 mmHg following orthostatic (postural) change [105, 106]. A sustained drop in blood pressure means blood is not exerting sufficient pressure on artery walls, preventing the brain and other organs from receiving adequate oxygen and nutrients [101]. Symptoms of OH may include fainting, palpitations, dizziness, lightheadedness, blurred vision, weakness, fatigue, nausea, headaches, shortness of breath, and pain in the chest, shoulder or neck [101]. Orthostatic hypotension often presents similarly to postural tachycardia, however, the former only occurs in the presence of orthostatic stress, whereas the latter can be attributed to both orthostatic and emotional stress [104].

### Prolonged QTc interval

The QT interval denotes an important component of the heart’s electrical activity on an electrocardiogram and represents part of the vital contraction and relaxation process of the cardiac muscle [107]. The QTc interval, more specifically, is a measure of the same time interval as the QT, however, has been corrected to account for the heart rate [108]. QTc prolongation can occur during an ED but may also suggest independent and reversible causes such as low potassium, calcium, glucose, or magnesium, or the use of psychotropic medications [109, 110]. A prolonged QTc interval can cause a life-threatening ventricular arrhythmia called torsade de pointes, ultimately resulting in ventricular fibrillation, which prevents the heart from pumping vital blood supply to the body [111].

## Electrolyte abnormalities

### Electrolytes

Electrolytes, or electrically charged particles or ions, are key regulators of the exchange of fluids, nutrients and waste products inside the body [68]. Electrolytes are lost in sweat during exercise and are negatively affected by purging behaviors, even at subclinical levels [65] [67, 112]. Common electrolytes affected in EDs are potassium, sodium and bicarbonate, with common symptoms including dizziness, weakness, fatigue, constipation, muscle pain, abnormal skin sensations and depression [65]. Electrolyte imbalances often underlie cardic abnormalities [113] and can result in rhabdomyolysis (the breakdown of muscle from strenuous exercise) and sudden death [65]. That said, many of the biochemical markers rectify with improved nutrition, meaning that exercise can gradually be modified with their improvement.

### Hypokalemia

Hypokalemia is a common electrolyte imbalance in ED, defined as low serum potassium. Potassium is a key electrolyte involved in normal cellular function, particularly in muscles and nerves [114]. Hypokalemia commonly presents in individuals with an ED engaging in purging behavior (BN or AN-P) and is prevalent in approximately 14% of individuals with BN [65]. Hypokalemia can also be triggered by malnutrition or the refeeding process [115]. Symptoms of hypokalemia may include muscle weakness, cramping, abnormal skin sensations, constipation, heart palpitations, fatigue, respiratory difficulty, and paralysis [65, 116]. Severe hypokalemia (< 2.5 mmol/L) can cause rhabdomyolysis, significant cardiac arrhythmias, and sudden death [65]. EKG changes due to hypokalemia can include “flat or inverted T waves, ST segment depression, and prominent U waves, which can exceed the amplitude of the T waves” [65]

### Hypophosphatemia

Hypophosphatemia occurs mainly in AN and is defined as low serum phosphate, often as a result of refeeding, malnutrition, chronic diarrhea, or overuse of diuretics or alcohol [113, 117]. Phosphate is a core component of the energy (adenosine triphosphate) used to produce muscular work [68]. Symptoms of low phosphate relevant to exercise include muscle dysfunction and weakness, abnormal blood oxygen levels, and irregular heartbeats [117]. Hypophosphatemia may also induce a state of confusion and delirium, anaemia, an increased severity of infections, rhabdomyolysis, coma, and even death [117]. Given the core role of phosphates in energy production, an individual experiencing these symptoms may warrant  restriction of high intensity activity and long durations of exercise engagement as these can result in an even greater drop in phosphate

### Hypomagnesemia

Hypomagnesemia, or low serum levels of magnesium, can result from diarrhea, diuretic use, hormone dysfunction, alcohol intake and some medications [116]. In the context of exercise, magnesium is involved in several processes, including oxygen uptake, energy production and the balance of electrolytes. Low magnesium can lead to muscle tremors and fasciculations, tetany, vertigo, eye problems, altered mental state, respiratory compromise, cardiac arrhythmias, ataxia, seizures, dysphagia, hormone and electrolyte disturbances, and sudden cardiac death [116]. Strenuous Exercise can induce a redistribution of magnesium in the body to accommodate metabolic needs, thus increasing the body's demand for magnesium 10-20%, may aggravate a current deficiency and increase risk of oxidative stress [118].

### Hypercarbia

Hypercarbia, also known as metabolic alkalosis, is a common clinical imbalance in people with ED [65] Similar to other electrolyte disturbances, hypercarbia can occur as a result of purging. Metabolic alkalosis is often asymptomatic; however, extreme cases may result in short and shallow breathing and respiratory complications [65]. Indicators of abnormal respiration, such as shortness of breath and signs of poor perfusion, including light-headedness, cyanosis, confusion, nausea, ataxia, pallor or cold and clammy skin [95]. During this state a break from resistance training and strenuous aerobic activity may be warranted until metabolic alkalosis has been resolved [119].

### Hyponatremia

Hyponatremia, a low concentration of serum sodium, may present with low blood volume status (a state where the body is left with lower than required levels of salt and water). Hyponatremia may occur due to purging or excessive sweating and is one of the most common electrolyte imbalances to occur in both AN and BN [65] Hyponatremia may also result from malnutrition (i.e., kidneys cannot excrete excess water into the urine, so excess water remains in the body), or it can present as a result of excessive water intake (most common in endurance sport activities) [60, 65]. Symptoms of hyponatremia include nausea, confusion, headaches, vomiting, delirium, impaired consciousness, seizures, and, in some cases, cardiorespiratory arrest [120]. For non-ED individuals, hyponatremia often occurs during or following a long or intense bout of activity, thus if currently in a hyponatrimic state due to an ED,  exercise should be stopped until rectified to reduced the risk of aggravating the condition [121].

## Biomedical function markers

### Temperature: hypothermia and hyperthermia

Hypothermia can occur as a direct result of malnutrition and hypoglycaemia [122], yet it can also persist beyond weight restoration [123]. Onset of hypothermia is related to decreased psychological and cardiac functioning, muscle rigidity, unconsciousness and death, and consequently, may warrant exercise modification until rectified [123].

Conversely, hyperthermia can result from failed thermoregulation, whereby the body produces or absorbs more heat than it can dispel through sweat. A serious medical complication of hyperthermia is heat stroke, which occurs due to excessive metabolic heat from intense exercise, excessive environmental heat, high humidity, some medications, and severe reactions to prescription medications [68]. Exercising in a hyperthermic state, especially if heat stroke presents, is a medical emergency as it can result in organ damage and ultimately mortality; thus, exercise in this state is contraindicated.

### Blood urea nitrogen and urine specific gravity

Blood Urea Nitrogen (BUN) and Urine Specific Gravity (USG) are markers of bodily functioning which may indicate diabetes, renal abnormalities, or overhydration, while high urine specific gravity can suggest adrenal, liver, heart or dehydration issues in individuals who are excessively sweating, vomiting, or who have diarrhea [124]. Exercise does not directly affect USG, however, both exercise and ED can impact hydration levels, and kidney and adrenal function, which are reflected in USG and BUN levels [124]. A negative balance in BUN indicates protein breakdown, which is harmful when it arises from muscle catabolism. Subsequently, it is recommended individuals only engage in strength training once a negative nitrogen balance is reversed, accompanied by consistent weight gain [78].

### Transaminase

Transaminase, a type of enzyme, is released into the bloodstream when the liver is damaged due to low weight, refeeding, alcohol intake, infection of the liver, or as a side effect of some medications [60, 113, 125, 126]. For indivdiuals with an ED, high levels of transaminase may result from the breakdown of bodily tissue (such as the liver) during severe low weight (BMI < 12) before refeeding [125] or due to the reduction of blood flow through the organs due to myocardial dysfunction [127], and can indicate vital organ failure [128]. During exercise, a significant increase in the rate of amino acid catabolism occurs, placing higher functional demands on the already compromised liver [129]. Thus, exercising with elevated transaminase levels (most often seen in AN) may cause additional harm and exercise intensity and type should be modified accordingly.

### Hypoglycaemia/hyperglycaemia

Hypoglycaemia is a state of low blood glucose and occurs commonly in individuals with AN [113, 130]. The presence of hypoglycemia can trigger a breakdown of both fat mass and FFM (i.e. muscle, bone, organs, etc.) in attempts to provide the brain with vital energy [60]. Exercising while hypoglycaemic harms the delivery of energy to the organs including the brain and working muscles [68]. Exercise can also worsen hypoglycaemia and its’ effects due to the high metabolic demand for glucose as energy during exercise [68]. The exacerbation of hypoglycaemia through exercise can result in coma and death, due to hepatic failure and the consecutive impairment in the process needed to increase glucose availability, gluconeogenesis [131, 132]. Thus, exercise may be contraindicated for individuals in a hypoglycaemic state until it has been resolved [68]. Individuals with BED may be at increased risk of hyperglycaemia or elevated blood glucose [133] Exercise engagement, especially high intensity or anaerobic exercise, can exacerbate hyperglycaemia, increase the risk of ketosis, or cause insulin dysregulation [134]. Indications for how to manage exercise prescriptions based on glucose levels in individuals with DM type 1 and 2 are available [134], however, there is a lack of research examining hyperglycaemia in individuals with ED.

### Dehydration (hypohydration)

Moderate exercise engagement over a one-hour period can produce a sweat loss of 0.5–1L [68]. Individuals who experience > 10% dehydration demonstrate a higher risk of developing tachypnoea or tachycardia, while those with 5–10% dehydration are at risk of developing peripheral edema [135]. Being severely dehydrated and unable to rehydrate contraindicates exercise as it may contribute to increased risk of heat illness, and negatively affect blood volume and exercise capacity [61, 65]. Mountjoy et al. (2014) note that dehydration-induced haemodynamic instability can contraindicate any sport engagement [57].

## Sex hormones

The following section relates to conditions of hormonal imbalances in EDs. The most effective method of preventing or reversing abnormal hormonal profiles is to increase energy availability above 30 kcal/kg of FFM/day, via either reducing exercise expenditure and/or increasing energy intake [136]. Adolescent females require greater energy availability than non-adolescent females to be able to continue normal menstrual function [137].

### Amenorrhea

Amenorrhea is the absence of a menstrual cycle for more than 90 days [138]. Functional hypothalamic amenorrhea (FHA), a subtype of amenorrhea, results from metabolic stress, including LEA or psychological distress [139]. Importantly, FHA can occur in the absence of weight loss [137]. Normal menstrual function may return with 6–12 months of weight stabilization; however, longer durations of amenorrhea may take longer to reverse [137]. Women with BED are also at elevated risk for both amenorrhea and oligomenorrhea, even when controlling for the occurrence of polycystic ovary syndrome [140]. Engaging in unmodified exercise while amenorrhoeic may detrimentally impact bone health and fertility [68, 141]. High impact exercise, such as jumping or running, without return to normalised sex hormones or resumption of menses, is contraindicated due to effects on long term bone health [142, 143]. Failing to modify exercise while amenorrhoeic may worsen existing hormonal and energy disruptions, further contributing to menstrual dysfunction and its’ consequences [137].

### Female sex hormones

Inadequate sex hormone concentrations, such as growth hormone-releasing hormone, luteinizing hormone, oestrogen and follicle-stimulating hormone, amongst others, can cause menstrual dysfunction, poor bone health, stunting of pubertal growth, infertility, and suboptimal muscular performance [137, 139, 144, 145]. Increasing energy availability and consistent weight gain can improve metabolic hormone profiles rapidly; however, exercise engagement must proceed cautiously while hormone profiles normalize [57].

### Male sex hormones

Sex hormones in males can be negatively affected in ED due to starvation-induced central hypothalamic hypogonadism [127]. Decreased concentrations of sex hormones, including testosterone, luteinizing hormone, follicle-stimulating hormone, and gonadotrophin-releasing hormone, as well as abnormal luteinizing pulsatility, can impair reproductive function, decrease immunity, and impact bone health [57, 127]. Similar to women, men with abnormal sex hormone profiles require increased energy availability and accompanying weight gain to normalize concentrations, and exercise engagement must occur cautiously to prevent further damage to bone, the stunting of pubertal growth, infertility, and suboptimal muscular performance [57].

## Body composition

### Body mass index

Body mass index (BMI) is a measure of body mass as a ratio of body weight to body height (kg/m^2^). BMI cannot solely or accurately depict health status; indeed, some individual’s weight is not affected at any stage of an ED despite the presence of severe illness, reflecting the need to evaluate the medical and psychological parameters of an individual’s health for a more accurate understanding [60]. However, BMI is still often used to help assess medical status in individuals with an ED, and a BMI < 75% of median values for age, sex, and height is suggested to indicate hospitalization [146]. Engaging in intense endurance or strength training exercise with a BMI < 13.5 kg/m^2^ may be contraindicated due to health and performance consequences from energy deficiency (e.g., increased risk of pericardial effusion and other effects of LEA; [59, 60]. Engaging in high impact activity < 16 kg/m^2^ [147], is suggested to be avoided due to the detrimental and widespread effects of LEA.

Appropriate exercise engagement may also be determined via progression of weight recovery considering an individual’s premorbid weight history [22, 148, 149]. For example, a weight loss of 5–10% in one month may contraindicate return to sport [57]. Another suitable marker of body composition is a prolonged low body fat percentage [measured by dual-energy X-ray absorptiometry (DEXA) or less accurately, anthropometry]. McCallum et al. also propose that prior to engagement in regular endurance or other rigorous training, an individual should restore to at least 90% of their target body weight [78].

### Low bone mineral density

Bone mineral density (BMD) is the amount of mineral density, particularly calcium and phosphorous, present in a volume of bone. Low BMD occurs when chronic LEA triggers a physiological adaptation within the bone to conserve energy for the vital organ systems [150]. Bone adaptations in the presence of chronic LEA include reduced oestrogen concentration, which is also involved in the disruption or elimination of reproductive function to conserve energy, as well as the suppression of some metabolic hormones [150]. Hormonal changes resulting from LEA can cause the uncoupling of bone turnover, whereby low levels of oestrogen permit increased bone resorption and, combined with the changes in metabolic hormones, cause decreased bone formation [150]. BMD and bone mineral geometry may be compromised most when accompanied by a low BMI, late menarche, or when amenorrhea persists for several years [151]. Although regular exercise engagement typically encourages bone growth [151], during a state of hypoestrogenism, exercise negates the usual positive effects of weight-bearing exercise [144].

Low BMD occurs in men and women, although sex differences may exist [152]. For example, one study suggests men with AN may display greater bone loss at the lumbar spine, as well as a faster rate of bone health decline, compared to women, with up to 65% of men experiencing low bone density as a symptom of their ED [152]. Once bone breakdown occurs, it may take months to years to recover following the resumption of adequate energy availability and recovered menses; however, in some cases, it may never fully recover [150, 153]. Consequences of poor bone health can include pain and disability, stress fractures and breaks, permanent rounding of the upper back due to micro fractures (kyphosis), and may require the use of a walker aid or pain medications, which can cause dependency and constipation, amongst other negative sequelae [60]. LEA prior to puberty may be particularly damaging to bone, as it may directly stunt both vital bone growth, as well as important sexual development that contributes to bone growth [154]. Recovery from bone growth delays can be challenging, and interruptions to bone accrual during adolescence and early adulthood can lead to osteoporosis and stress fractures [151].

Howgate and colleagues recommend high impact activity may be contraindicated until a BMI of 16 kg/m^2^ is reached to prevent compromise to bone health [147]. Furthermore, unmodified exercise in the presence of LEA, amenorrhea, and low BMD increases one’s risk of bone stress injury and stress fractures [57, 155]. Miller and colleagues (2006) found that in those with an ED that the greatest increases in lumbar spine and hip bone densityoccurred in patients who increased their body weight to 85% IBW and resumed menses (having had at least one menses in the past three month). Importantly, they found that increase in FFM was a better determinate improve BMD than adipose tissue gain highlighting the importance of resistance training for this group [156]. Thus, if low BMD presents in the absence of low BMI, malnutrition, LEA, or sex hormone disturbances. strength training can improve low BMD in men and women [157].

### Superior mesenteric artery syndrome

Superior mesenteric artery (SMA) syndrome is caused by the compression of the duodenum due to a diminished fat pad from weight loss that would usually separate the aorta and superior mesenteric artery [127]. Symptoms of SMA syndrome can include upper quadrant abdominal pain soon after eating, early satiety, nausea, and vomiting [127]. SMA is most commonly found in children or individuals with a low BMI and can resolve with weight gain [158, 159]. However, as SMA syndrome occurs in severly underweight individuals, it may be prudent to modify exercise during this time.

### Peripheral edema

Peripheral edema is the retention of fluid, most commonly in one’s extremities, and is particularly prominent in individuals with AN [160]. Higher intensity exercise can increase edema, which may already be aggravated during refeeding, or due to renal filtration of insulin or due to high levels of carbohydrate consumption [21]. Thus, exercise may be contraindicated in the presence of peripheral edema [21].

## Exercise contraindication, modification, or with caution in the context of EDs

Recommendations for exercise modifications and exercise contraindications prior to exercise engagement are summarized in Table 2. Interestingly, some of the medical complications and associated exercise recommendations were documented through non-ED research publications, and research guiding modification of specific times, types, and intensity of exercise in the presence of each complication or contraindication is lacking. Table 2 highlights important gaps in the research literature on ED and exercise duration, type, intensity, and frequency to be able to provide science-based exercise recommendations.

**Table 2:** Summary of exercise recommendation evidence

Symptom	Recommendation	Population
*Energy availability*		
Low energy availability	Modify	ED
Starvation	* H/I A & AN contraindicated on days when meals are skipped	ED
Purging	*Contraindicated when hypovlemia is present	ED
*Cardiovascular health*		
Arrhythmias	EKG, Holter monitoring and exercise testing if arrhythmias persist beyond nutritional restoration	GEN
Bradycardia	Only supervised & with caution (particularly if under 80% IBW, and purging)	ED
Tachycardia	Investigate cause prior to engagement	GEN
Postural tachycardia	Modify	GEN
Orthostatic hypotension	Unknown	N/A
Hypotension	Modify until resolved	ED
Hypertension	High intensity & volume contraindicated for BP > 200 mm/Hg)	GEN
Prolonged QT	Unknown	N/A
*Electrolyte abnormalities*		
Hypokalaemia	Unknown	N/A
Hypophosphatemia	High intensity & long duration activity contraindicated until rectified	ED
Hypomagnesemia	High intensity exercise restricted until rectified	GEN
Hypercarbia	High intensity aerobic and resistance training restricted until rectified	GEN
Hyponatremia	Correct prior to long or intense activity	GEN
*Biomedical function markers*		
Hypothermia	Modify	GEN
Hyperthermia	Contraindicated	GEN
Blood urea nitrogen & urine specific gravity	*Strength training only when nitrogen levels are normalized	ED
Transaminase	Modify type and intensity	GEN
Hypoglycaemia	Contraindicated until resolved	GEN
Hyperglycaemia	Exercise with caution, contraindicated when > 250 mg/dl	GEN
Hypohydration	Contraindicated	ED
*Sex hormones*		
Amenorrhea/functional hypothalamic amenorrhea	Modify *high impact exercise is contraindicated in those with FHA	ED
Low female sex hormones	Exercise with caution	ED
Low male sex hormones	Exercise with caution	ED
*Body composition*		
Body mass index (kg/m^2^)	Modify *high impact exercise is contraindicated when BMI < 16 kg/m^2^ *intense endurance or strength training contraindicated when BMI < 13.5 kg/m^2^	ED
Bone mineral density	Activity dependent	ED
Superior mesenteric artery syndrome	Modify until resolved	ED
Peripheral edema	High intensity contraindicated	ED

BMI: body mass index; ED: diagnosed eating disorder sample; GEN: general population; *specific recommendations; DM: Diabetes Mellitus; H/I A& AN: High intensity aerobic and anaerobic exercise; H/I & Vol – High intensity and volume; FHA: Functional hypothalamic amenorrhea; BP: blood pressure; IBW: Ideal Body Weight

## Implications for practice

Given the serious concerns for safety with exercise among medically and physiologically compromised individuals with EDs, ED treatment teams and practioners understandably tend to recommend abstinence from exercise during ED treatment. However, research over the last two decades has demonstrated that complete restriction from exercise is not necessary or appropriate in many cases, and may actually do more harm than good [161]. The purpose of the current review was to provide a comprehensive summary of the medical and physiological complications of EDs that may be exacerbated among individuals who engage in dysfunctional exercise, and to offer an initial set of recommendations for incorporating exercise during ED treatment based on our findings. We have summarized the complications and recommendations in Table 1 and Table 2, respectively, to serve as a resource for members of ED treatment teams to help evaluate more readily and confidently whether exercise is safe for individual patients and when modifications and caution may be warranted. The information provided from this review may also be used in conjunction with the Safe Exercise at Every Stage guideline to help ED clinicians better address and manage dysfunctional exercise during treatment [165]. Ultimately, it is our hope that this knowledge will further empower clinicians in this challenging area of ED treatment.

## Implications for future research

Our review highlights some critical gaps in the literature on exercise in ED contexts. There was limited evidence for guiding specific recommendations for exercise duration, type, intensity, and frequency in the presence of each complication. This includes a lack of differentiation between the mechanical versus metabolic loading of exercise and how these may uniquely impact the safety of a given exercise. For example, recommendations based upon exercise “intensity” alone may be misleading as they do not account for the differential demands of high mechanical and low metabolic loading versus low mechanical and high metabolic loading. This information is needed to inform the development of guidelines to best assess and manage exercise in ED populations [17]. Future research must prioritise increasing our knowledge of exercise as well as further adaptations relevant for special ED populations, such as athletes, youth, or individuals with comorbid conditions such as diabetes mellitus.

Most clinicians treating ED have not received adequate training in exercise interventions [162], with many highlighting their lack of knowledge and fears surrounding exercise as reasons for which they avoid addressing exercise with their clients [163]. Therefore, the inclusion of an exercise professional (e.g., accredited exercise physiologist, athletic therapist) with specialized training in ED as part of the treatment team may offer a depth of knowledge surrounding exercise physiology not otherwise available [162]. Even with this addition, determining readiness to return to exercise for those with ED has long been debated, with little evidence-based research currently guiding treatment [164]. As mentioned above, guidelines such as the Safe Exercise at Every Stage (SEES) Guideline [165]for adults with an ED [165], or SEES-Athlete version or RED-S CAT for athletes with an ED [166, 167] may provide guidance for practitioners aiming to determine physical and psychological readiness for exercise engagement.

## Conclusion

This narrative review outlined the medical and physiological complications of EDs which can be negatively exacerbated for individuals who engage in dysfunctional exercise. These complications should be monitored in individuals with EDs to determine whether exercise is contraindicated, or whether it can be used in a modified or cautionary way. Importantly, if exercise in ED recovery is nutritionally supported and safely modified, it can facilitate ED treatment and recovery, and bring health benefits to individuals living with an ED [17, 168, 169]. Clinicians working with individuals with ED require clear guidelines to aid decision-making as it relates to exercise engagement during treatment.

## Data Availability

All data generated or analysed during this study are included in this published article [and its supplementary information files].

## Abbreviations

AN-P: Anorexia nervosa purging subtype
AN: Anorexia nervosa
BED: Binge eating disorder
BN: Bulimia nervosa
BMD: Bone mineral density
BMI: Body mass index
Bpm: Beats per minute
BUN: Blood Urea Nitrogen
DEX: Dysfunctional exercise
DEXA: Dual-energy X-ray absorptiometry
EDNOS: Eating disorder not otherwise specified
ED: Eating disorders
FFM: Fat free mass
FHA: Functional hypothalamic amenorrhea
Kcal: Kilocalories
Kg: Kilogram
LEA: Low energy availability
OEA: Optimal energy availability
RMR: Resting metabolic rate
SMA: Superior mesenteric artery
VO_2_max: Maximal oxygen uptake
USG: Urine Specific Gravity
